# Management of Subglottic Stenosis With the “Maddern Procedure”: Long Term Follow‐Up Outcomes

**DOI:** 10.1002/lary.70032

**Published:** 2025-08-20

**Authors:** Emilie A. C. Dronkers, Gemma M. Clunie, Aphrodite Iacovidou, S. A. Reza Nouraei, Chadwan Al Yaghchi, Guri S. Sandhu

**Affiliations:** ^1^ National Centre for Airway Reconstruction Imperial College Healthcare NHS Trust London UK; ^2^ Department of Otorhinolaryngology/Head and Neck Surgery Leiden University Medical Center Leiden the Netherlands; ^3^ Department of Otolaryngology Nottingham University Hospitals NHS Trust Nottingham UK

**Keywords:** airway reconstruction, airway stenosis/reconstruction, airway stenosis—clinical, otolaryngology, otorhinolaryngology

## Abstract

**Objective:**

Full‐thickness resection and skin grafting of the laryngotracheal junction, the “Maddern Procedure,” can be used to treat patients with recalcitrant laryngotracheal stenosis. We report the long‐term surgical outcomes of this procedure.

**Methods:**

A consecutive series of 27 females with idiopathic, iatrogenic, or vasculitic subglottic stenosis who underwent the Maddern procedure between 2012 and 2023 were retrospectively reviewed. Airway outcome was assessed using the Modified Medical Research Council Dyspnea (mMRC) Scale. Secondary outcomes included voice, swallowing, mucus management, complications, and the need for subsequent airway surgery.

**Results:**

Most patients had idiopathic subglottic stenosis (*n* = 20, 74.1%), followed by vasculitic (*n* = 4, 14.8%) and intubation‐related (*n* = 3, 11.1%) etiologies. Patients had an average of 7.3 (SD 3.4) endoscopic procedures before undergoing the Maddern procedure. Mean pre‐Maddern inter‐treatment interval was 7.5 months (SD 3.4). The median follow‐up was 6.6 years (interquartile range 0.75–11.3). The mMRC dyspnea scale improved from 2.7 (SD 0.73) to 0.52 (SD 0.85). The five‐year recurrence‐free rate was 63%. It differed between etiologies, ranging from 83% for idiopathic stenosis to 67% for intubation‐related and 0% for vasculitis‐related stenoses. During the first 4 years after Maddern, 67% of patients reported daily cough and the need to use nebulizers regularly.

**Conclusion:**

The Maddern procedure is an effective and durable option for patients with idiopathic subglottic stenosis. Careful screening of patients with presumed idiopathic subglottic stenosis for possible underlying auto‐immune conditions remains important when considering patients for this procedure.

**Level of Evidence:**

Level 4.

## Graphical abstract

The Maddern procedure is an effective and durable option for patients with idiopathic subglottic stenosis. Five‐year recurrence‐free rate is 83%, and the mMRC dyspnea scale improves from 2.7 (SD 0.73) to 0.52 (SD 0.85).

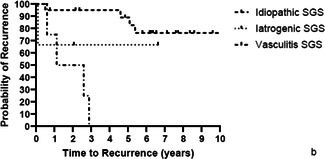

## 1 Introduction

Acquired subglottic stenosis (SGS) is a rare condition characterized by narrowing of the airway below the vocal folds due to fibrotic tissue. It can be caused by a range of conditions, from iatrogenic causes such as intubation trauma to a variety of inflammatory and auto‐immune conditions such as granulomatosis with polyangiitis (GPA) [1, 2]. Idiopathic subglottic stenosis (iSGS) is a rare, slowly progressive, fibro‐inflammatory process of unknown cause that leads to narrowing of the airway in the subglottic region and usually involves the cricoid and first tracheal rings. The disease is seen predominantly in females with European ancestry and usually after puberty [3]. The disease involves the mucosa and submucosa, sparing the cartilage of the cricoid and trachea, and tends to recur even after repeated endoscopic dilatations [4]. Cricotracheal resection has a higher level of success but tends to lower the pitch in these young to middle‐aged women and still has a 10%–15% chance of long‐term recurrence (> 10 years) [5, 6]. The “Maddern procedure,” resection of the subglottic scar by replacing the resected mucosa with an autologous skin graft, was developed by the senior author in 2012 following a patient's request to find a definite solution without a scar on the neck [7]. With this procedure, the diseased mucosa is resected while leaving the unaffected cartilaginous framework intact, and the mucosa is reconstituted with a split‐thickness skin graft (SSG) to prevent restenosis and optimize wound healing. The use of skin grafting to inhibit scar formation had already been described in the management of Dupuytren's disease, and skin in airway reconstruction was first used by M F Arbuckle in St Louis in 1927 [8].

In this article, we describe the long‐term surgical outcomes of the Maddern procedure as treatment for subglottic stenosis.

## 2 Materials and Methods

All patients with subglottic stenosis who underwent the Maddern procedure between 2012 and 2023 at the National Centre for Airway Reconstruction at Charing Cross Hospital, Imperial College Healthcare NHS Trust (ICHT), London, UK, were consecutively included in this descriptive retrospective case series. Service evaluation approval was given by ICHT (reference ENT 408), and governance was provided via the ICHT Service Evaluation and Audit committee. All patients experienced longstanding and recalcitrant subglottic stenosis from either idiopathic, iatrogenic, or vasculitic etiology. They were referred to the UK's National Centre for Airway Reconstruction for tertiary care. Patients were considered for the Maddern procedure when their subglottic stenosis proved refractory to endoscopic dilation—including shortening treatment intervals—or if patients wished for more permanent management without the need for an open resection. As the technique was developed by the senior author, there was a learning curve for the right surgical indication based on outcomes of previously treated patients.

Diagnosis of subglottic stenosis was confirmed with microlaryngoscopy, biopsy, CT‐scan, and serum analysis for ANA, ANCA, and rheumatoid factor. Etiology of subglottic stenosis was classified based on biopsy and blood serum results confirming vasculitis, or having a history of trauma or traumatic or prolonged intubation confirming iatrogenic cause, or else idiopathic if the first causes were ruled out. Patients with multi‐level airway stenosis were not eligible for the Maddern procedure; initially, neither were patients with a stenosis < 10 mm from the true vocal fold edge, inadequate endoscopic visualization, BMI > 35, or other comorbid diseases that lead to an increased anesthetic risk for prolonged supraglottic jet ventilation.

Clinical and demographic characteristics of included patients were retrospectively collected from electronic medical records. Variables included were: treatment history before and after Maddern procedure, operative details, complications classified by Clavien‐Dindo grade [9], and need for subsequent procedures for either mucous management or recurrent stenosis. Additionally, specific functional outcomes were extracted, including: Modified Medical Research Council Dyspnea Scale (mMRC) [10], and narrative details on voice, swallowing, and mucous management. The mMRC scale is used to assess the degree of baseline functional disability due to dyspnoea: a score of 0 refers to dyspnoea with strenuous exercise, and a score of 4 relates to being too dyspnoeic to leave the house or breathless when dressing.

Follow‐up was defined as the time in years between the Maddern procedure and last patient contact. Recurrence‐free rate was defined as the time to the first surgical procedure post‐Maddern to address recurrent subglottic stenosis—including endoscopic dilation, serial intralesional in‐office steroid injection (SILSI) and open resection—or last patient contact.

### 2.1 Surgical Technique

All Maddern procedures in this study were performed by the senior author (G.S.) under general anesthesia. The procedure was performed as described in detail by the senior authors in previous publications [7, 11]. Video 1 visualizes the full procedure. Only one patient in this series had a temporary tracheotomy during the procedure; the other patients were all ventilated using supraglottic high frequency jet ventilation.

**VIDEO 1:** Video of Maddern procedure. Video content can be viewed at https://onlinelibrary.wiley.com/doi/10.1002/lary.70032. [Color figure can be viewed in the online issue, which is available at www.laryngoscope.com]

**FIGURE 1:**
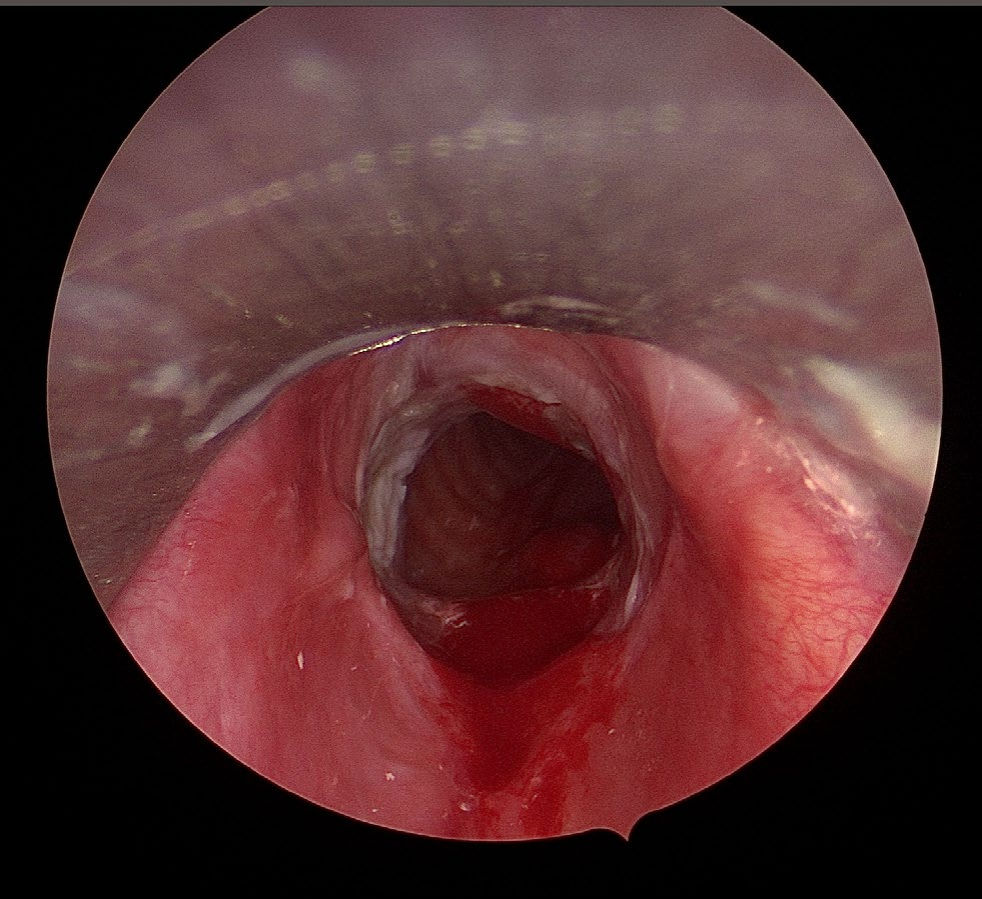
Keratin build‐up 6 weeks post‐Maddern procedure. [Color figure can be viewed in the online issue, which is available at www.laryngoscope.com]

Resection of full thickness subglottic scar down to the level of cricoid and first tracheal ring perichondrium was performed using a microdebrider, after determining the depth to the underlying cartilage using CO_2_ laser assisted radial cuts. Resection of scar tissue overlying trachealis was performed very carefully as to prevent the creation of an inadvertent tracheoesophageal fistula. An endoluminal stent, fashioned from a silastic T‐tube limb size 10–12, cut to the size of the stenotic segment, was covered with a SSG from the thigh and sutured in place with the dermis facing outward with a layer of paraffin impregnated gauze between skin and stent. The stent was then sutured endoscopically to the neck; this stay suture could be removed 2 weeks after. Postoperatively, patients were admitted to a highly specialized otolaryngology ward; saline nebulizer (four times a day) was utilized for 2 weeks to limit secretion buildup within the stent. Preventive antibiotics were prescribed with Co‐Amoxiclav (625 mg, three times per day) for the first 7 days followed by Ciprofloxacin (500 mg, 2 times a day) for the next 7 days to prevent Pseudomonas infection. A “second look” microlaryngoscopy procedure was performed as a standard 4–6 weeks later to laser any excess keratin debris and granulation tissue (Figure 1).

### 2.2 Statistics

Statistical analyses were performed using GraphPad Prism (v9.3.1). Descriptive statistics, including Pearson's chi‐square test for categorical data and student's *t*‐test and Wilcoxon rank test for continuous variables, were performed. Kaplan–Meier survival curve generation and log‐rank analysis were used to test for recurrence‐free rate. All tests were two‐sided, and *p*‐values below 0.05 were considered statistically significant.

## 3 Results

Between 2012 and 2023, *n* = 27 patients underwent the Maddern procedure at the National Centre for Airway Reconstruction. All patients were female, and the mean age at the time of surgery was 47 years (SD 12.1, range 24–69). Most patients had idiopathic SGS (74.1%), followed by vasculitic SGS (14.8%) and iatrogenic SGS (11.1%). Two patients with ANCA‐positive vasculitis were compliant with immunotherapy at the time of the Maddern procedure. The other two patients were diagnosed 1–3 years after their Maddern procedure, as prior to the procedure, all their biopsies and blood results came back as ANCA‐negative. Table 1 shows the demographics of all patients. None of the patients had a tracheotomy at the time of the Maddern procedure.

**TABLE 1:** Demographics of all patients with subglottic stenosis (*n* = 27).

All patients		*N* (%)	Mean (SD)
Age at diagnosis (years)			41.1 (11.8)
Etiology of SGS	Idiopathic SGS	20 (74.1%)	
	Iatrogenic SGS	3 (11.1%)	
	Vasculitis SGS	4 (14.8%)	
Number of pre‐Maddern endoscopic dilations			7.6 (3.4)
Mean interval between procedures pre‐Maddern (months)			7.5 (3.4)
Pre‐operative mMRC dyspnea scale			2.7 (0.73)
Post‐operative mMRC dyspnea scale			0.52 (0.85)
Age at Maddern (years)			46.9 (12.1), range 24–69
Number of post‐op keratin clean up procedures			0.93 (1.14)
Number of patients with recurrent stenosis within 10 years after Maddern	Yes	11 (41%)	
	No	16 (59%)	
Number of post‐Maddern surgical procedures for recurrence			1.3 (2.1)
Mean interval between procedures post‐Maddern (months)			14.6 (8.1)

The mean number of pre‐operative endoscopic dilations was 7.3 (SD 3.4) with a mean pre‐operative treatment interval of 7.5 months (SD 3.4). Overall, patients had a mean interval of 6 years between diagnosis of subglottic stenosis and the Maddern procedure. In the group of idiopathic SGS patients, there was a shorter interval of 4 years. The median follow‐up time in this retrospective study was 6.6 years (range 0.75–11.3 years) Three patients had a follow‐up of less than 2 years, and three had a follow‐up of over 10 years. Overall, *n* = 11 patients (41%) had a recurrent operation during this follow‐up time.

### 3.1 Functional Outcome of Breathing

**FIGURE 2:**
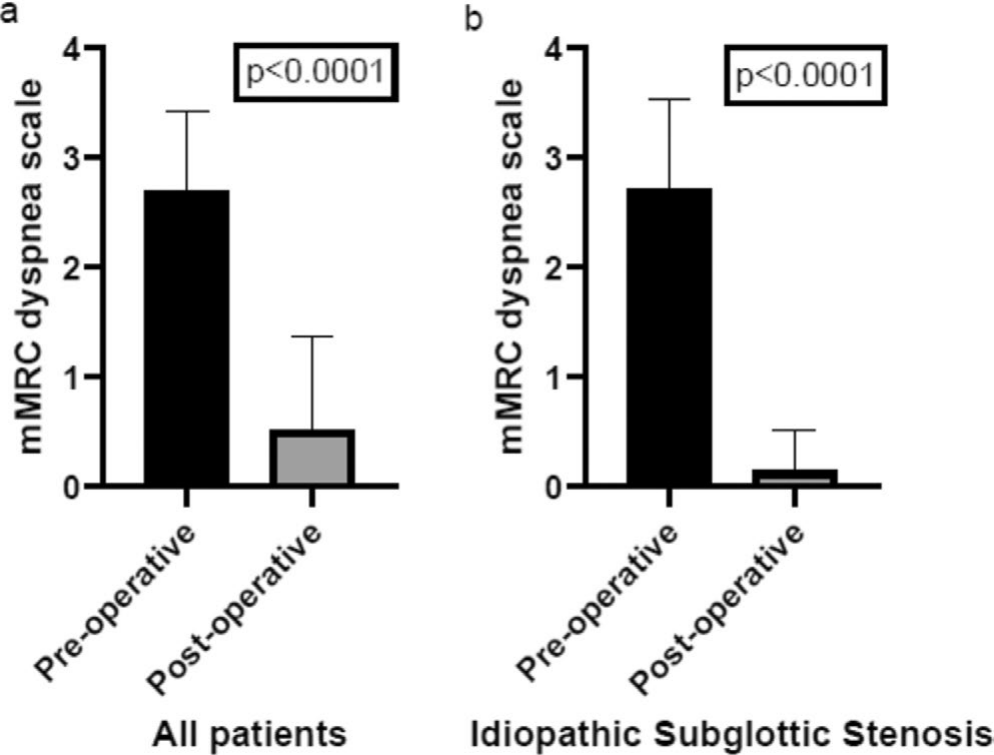
(a, b) Histogram of Preoperative and Postoperative mMRC Dyspnea Index Scores. (a) All included patients with subglottic stenosis. (b) Patients with idiopathic subglottic stenosis. MMRC, modified medical research council.

The Maddern procedure was successful in terms of improved breathing in all 27 patients during their first post‐operative consultation 4–6 weeks after stent removal. The mMRC dyspnea scale improved significantly (*p* < 0.0001) from pre‐operative mean score of 2.7 (SD 0.73) to post‐operative mean score of 0.52 (SD 0.85), as shown in Figure 2a. The post‐operative mMRC dyspnea score in patients with idiopathic SGS was even better at 0.15 (SD 0.37) (Figure 2b). Table 2 shows all outcomes for this specific subgroup of patients.

**TABLE 2:** Outcomes of Maddern procedure for patients with idiopathic subglottic stenosis (*n* = 20).

Idiopathic SGS	*N* (%)		Mean (SD)
Age at Maddern (years)			50.5 (11.6) range 24–69
Number of pre‐operative dilations			7.4 (9.8) range 2–23
Pre‐operative mMRC scale			2.7 (0.83)
Interval between pre‐op dilations (months)			7.4 (3.3) range 3–14
Post‐operative mMRC scale			0.15 (0.37)
Number of post‐op keratin clean up procedures			0.9 (1.07)
Number of patients with recurrent stenosis within 10 years after Maddern	Yes	6 (30%)	
	No	14 (70%)	
Number of post‐op dilation procedures for recurrence^a^			0.65 (1.2)
Mean interval between procedures post‐Maddern (months)^b^			18 (7.2)

^a^ For *n* = 6 patients with recurrent stenosis.

^b^ For *n* = 3 patients with idiopathic SGS with multiple recurrences after Maddern.

### 3.2 Recurrence Free Rate

**FIGURE 3:**
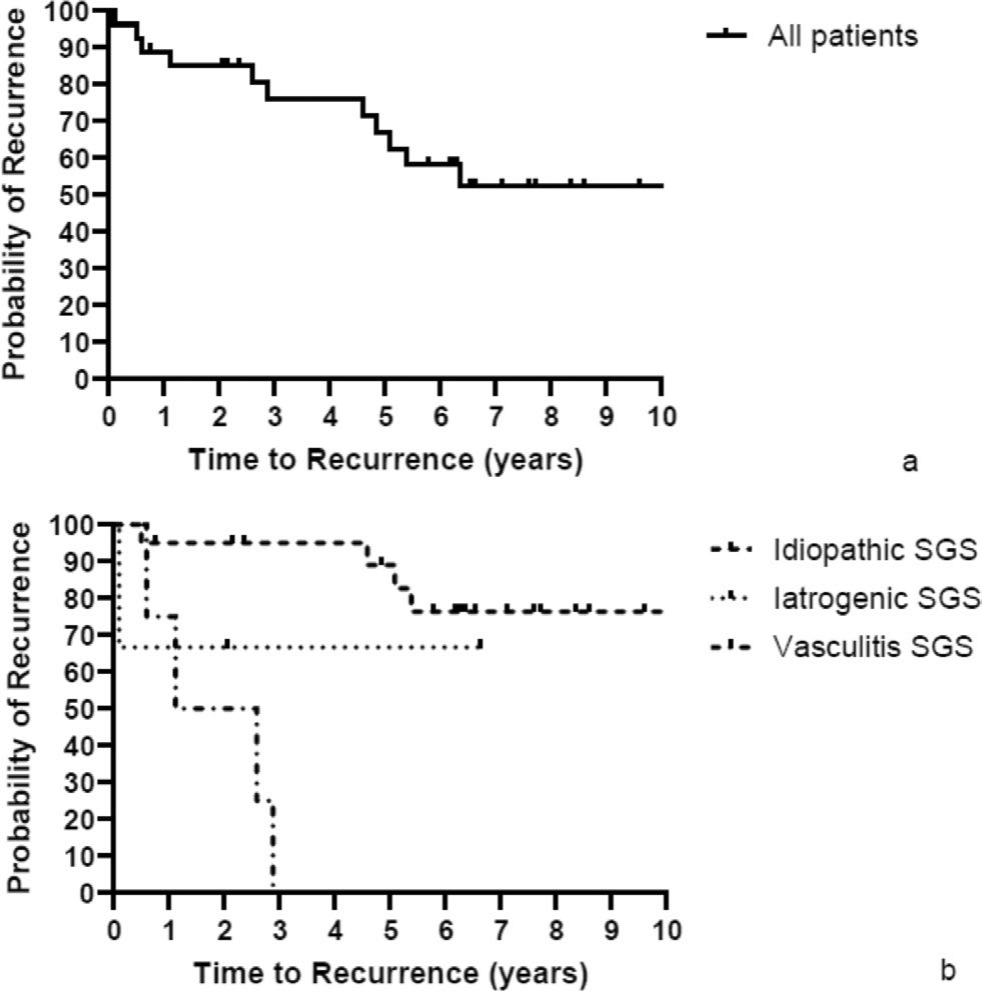
(a) Need for and time to surgical procedure to manage recurrence of subglottic stenosis post‐Maddern procedure. (b) Need for and time to surgical procedure to manage recurrence of subglottic stenosis post‐Maddern procedure per etiology.

Figure 3a illustrates the time to surgical procedure to manage recurrent SGS post‐Maddern procedure for all patients, and Figure 3b shows how these rates differ across etiologies of SGS. In Table 3 the recurrence‐free rates are shown per etiology. Overall, the 5‐year recurrence‐free rate was 63%. Patients with idiopathic subglottic stenosis had a significantly (*p* < 0.0001) more favorable outcome of the Maddern procedure both in the short and long term than patients with iatrogenic or vasculitic etiology. The 5‐year recurrence‐free rate for patients with idiopathic SGS was 83%. All patients with a recurrent stenosis were managed with either endoscopic dilation, SILSI, or a combination of these. None of the patients with a recurrent stenosis has proceeded with open reconstructive surgery.

**TABLE 3:** Recurrence free rates.

	All patients (*n* = 27)	Idiopathic SGS (*n* = 20)	Iatrogenic SGS (*n* = 3)	Vasculitic SGS (*n* = 4)
2‐year recurrence‐free rate	85%	95%	67%	50%
5‐year recurrence‐free rate	63%	83%	67%	0%
10‐year recurrence‐free rate	52%	76%	67%	0%

### 3.3 Complications

There were two patients with Clavien‐Dindo grade 3b post‐operative complications who required surgical intervention under general anesthesia. In one patient, the Maddern procedure was complicated by a small tracheoesophageal fistula, which was discovered during stent removal 2 weeks later. The fistula was successfully repaired with a smaller tracheal resection technique and primary closure during an open procedure 1 month later, and the patient did not have any long‐term morbidity. The fistula was thought to be related to more aggressive microdebridement at the level of trachealis. As she was one of the first patients to undergo the Maddern procedure, very careful resection of scar tissue at the trachealis level has been practiced since. One patient suffered from subglottic granulation tissue requiring steroid injection 6 weeks after Maddern. This granulation tissue developed at the distal end of the stent. This patient was also one of the first to undergo Maddern, and more attention has been paid to fashioning the SSG with overhang around the stent since. There were no patients with post‐operative wound complications, and there were no Clavien‐Dindo grade 4/5 complications (life‐threatening complications, death). Two patients (7.4%) had minor voice problems. One of them had a pre‐existent left vocal cord palsy; the other developed a polyp of the left vocal cord and difficulty projecting her voice 7 years after Maddern. This was thought to be related to a Covid‐19 infection. None of the patients reported issues with swallowing or aspiration post‐Maddern.

### 3.4 Mucus Management

All patients struggled with mucus management due to increased production of phlegm and the need to frequently cough and nebulise prior to the Maddern procedure, as is known to be normal for this patient group [12]. Due to SSG being used around the stent that covers the tracheal wall, patients are at risk of developing excess keratin debris in their subglottis and trachea, which can increase the amount of mucus related to the absent ciliary clearance in this area.

Daily issues with mucous management are quite common, with 67% (*n* = 18) of all patients reporting daily coughing and requiring frequent nebulizing with a 0.9% saline solution in the first 4 years after Maddern. Of those patients, 72% (*n* = 13) needed further surgical management, a microlaryngoscopy with CO_2_ laser evaporation of excess keratin buildup, to manage mucus. They had a mean of 1.9 keratin clean‐up procedures (SD 0.8), excluding the standard second look procedure 4–6 weeks after stent removal. Most were in the first 2 years after Maddern. After 4 years, none of the patients needed further clean‐up procedures for mucus management, although 22% (*n* = 6) of all patients reported an ongoing need for daily nebulizing to handle mucus and cough > 5 years after the Maddern procedure.

## 4 Discussion

This study describes the largest case series of the Maddern procedure for patients with subglottic stenosis reported in the literature to date and has the longest follow‐up of up to 11 years with a median of 6 years. As the Maddern is an innovative technique, and only two previous studies [11, 13] have published on initial results for, respectively, *n* = 9 and *n* = 26 patients, the current study finally allows us to draw conclusions on the long‐term outcomes.

This study shows that the Maddern procedure has favorable results in patients with a background of idiopathic subglottic stenosis with only 5% recurrence in the first 2 years, a 5‐year recurrence rate of 17%, and a 10‐year recurrence rate of 24%. Out of the three patients with an iatrogenic etiology, only one developed a single re‐stenosis requiring surgical management. As this was only a small subgroup of the study, it is difficult to extrapolate the results and suggest the effectiveness of the Maddern procedure in patients with an iatrogenic subglottic stenosis. Before the development of the technique as described in this paper, using laser to endoscopically resect all scar tissue in both idiopathic and iatrogenic stenosis cases led to failure. Applying it to idiopathic SGS with “cold steel” debridement of the stenosis produced an unexpected favorable outcome. This may be related to the fact that idiopathic SGS generally has a good blood supply when compared to mature scar.

All patients with vasculitic origin of SGS developed recurrent stenosis within 3 years after the Maddern procedure. Another recent study [13] on the Maddern procedure showed the results of four patients with ANCA positive vasculitis, and none of these developed a recurrent stenosis requiring further surgical treatment post‐Maddern. However, in this series all patients were already diagnosed with ANCA positive vasculitis prior to their Maddern procedure, were compliant with immunotherapy regimens, and were at least 1 year after their last inflammatory flare. In our series, two patients were diagnosed 1–3 years after their Maddern procedure, as prior to the procedure all their biopsies and blood results came back as ANCA negative. They started on immunotherapy after the Maddern procedure and have been managed with endoscopic dilation and SILSI since. The other two patients were initially compliant with immunotherapy but developed re‐stenosis shortly after they stopped the medication. Based on the results of both studies, more research is needed regarding the role of immunotherapy in patients with ANCA positive vasculitis being treated with the Maddern procedure. Also, careful screening of patients with presumed idiopathic subglottic stenosis for possible underlying auto‐immune conditions remains important when considering the Maddern procedure.

According to the latest North American Airway Collaborative (NoAAC) publication [3]. On their multi‐institutional prospective cohort study regarding surgical treatment options in 487 patients with idiopathic SGS, cricotracheal resection (CTR) has the lowest rate of recurrence requiring surgical intervention (5%) in their 5‐year follow‐up study. Cricotracheal resection is often considered in patients with subglottic stenosis because it is described as the most curative and durable surgical option in the literature [5, 14, 15, 16]. Yet, the inherent risks of open reconstruction, along with the decrease in fundamental frequency of 10–50 Hz related to cricothyroid muscle damage [17], make it a less attractive option [18, 19, 20], especially for the (female) population with idiopathic subglottic stenosis. The Maddern procedure has a 5‐year recurrence rate of 17% in our study, and 22% and 27% in previous publications on this technique [11, 13]. In contrast to CTR, healthy cartilage is preserved with the Maddern procedure. In a mucosal disease such as idiopathic subglottic stenosis, avoidance of the resection of normal cartilage is optimal. Long term (> 10 year) follow‐up data on CTR for idiopathic SGS are scarce but show a 10%–15% recurrence [5]. Revision CTR is a potential option for patients, but remains challenging as there is a maximum to the length of tracheal cartilage that can safely be resected and anastomosed. The Maddern procedure, on the other hand, can be repeated many times, and is also a possibility for revision surgery after failed CTR.

Endoscopic resection with adjuvant medical therapy (ERMT) has also been mentioned as a more durable alternative to endoscopic dilations, with a 5‐year recurrence rate of 30% [4]. Comparable to the Maddern procedure, there is no increased risk of changes to the voice. However, the Maddern procedure has a more durable effect with lower recurrence rates. Also, in contrast to ERMT, there is no need to adhere to a 2‐year adjuvant medical treatment program including long‐term preventive antibiotics, a steroid inhaler, and a proton pump inhibitor (PPI) [21] although most patients do need to nebulize daily with a saline solution for the first 2 years.

**FIGURE 4:**
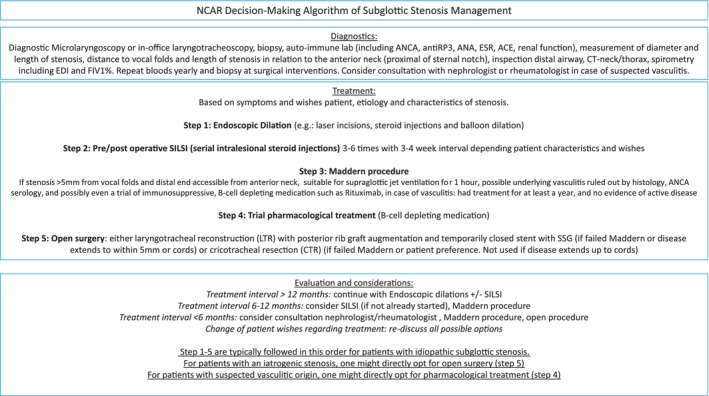
National centre for airway reconstruction decision‐making algorithm of subglottic stenosis management. [Color figure can be viewed in the online issue, which is available at www.laryngoscope.com]

Based on the extensive clinical experience in our center with the Maddern procedure, treatment of subglottic stenosis in general, and the results of the current study, the authors would suggest adding this technique to the surgeons' armamentarium for treatment of subglottic stenosis as shown in Figure 4.

There are two major limitations to the current study. The retrospective nature of the case series and the lack of pre‐ and postoperative standardized outcomes and Patient Reported Outcome Measures (PROMs) withhold us from drawing objective conclusions on functional outcomes. A prospective study with pre‐ and postoperative airflow measures and use of a core outcome set for patients with SGS, including instrumental measurement of voice, swallowing and PROMs would be helpful [22]. The inclusion criteria for patients to be considered for Maddern procedure in this study were quite clear, but have evolved over time, which is inherent to the maturation of an innovative surgical technique. Patients with a confirmed or possible underlying vasculitis are now very cautiously being considered for this technique. Also, patients with a stenosis between 5–10 mm distal from the vocal folds are now being considered for this technique as there is still some space for the open stent to sit conveniently in the trachea without the need for a transglottic extension of the stent and subsequent risk for further scar and structuring due to direct contact of the stent with the vocal folds. Although technically feasible, the Maddern procedure has not been offered for cases where the disease extends up to the vocal folds, especially at the posterior commissure. For patients with posterior glottic scar the authors still prefer a laryngofissure approach, with posterior cricoid split, augmentation using costal cartilage and a temporarily SSG covered, closed, transglottic stent, that has a high level of success in these more complicated cases of stenosis where glottic disease frequently leads to impaired vocal fold function [23]. Finally, the data reflect the experience of a single surgeon and inventor of the technique. Moreover, all patients were referred to our tertiary center as they were challenging to manage surgically and had multiple endoscopic dilations prior to their Maddern procedure. This may have induced selection bias, and based on the results of the current study it is yet unclear what the role of the Maddern procedure could be in patients with newly diagnosed and not previously treated subglottic stenosis.

Future research should focus on optimizing the graft material. Our study shows that 67% of patients struggle with coughing and need to nebulize with saline solution for the first 4 years following the Maddern procedure. Almost half of all patients (48%) needed a microlaryngoscopy clean‐up procedure to resurface excess keratin build up, and 22% (*n* = 6) of all patients reported an ongoing need for daily nebulizing due to mucus and cough even 5 years after Maddern. Exploration of other management options for mucus and cough would be beneficial alongside the development of the surgical technique [12]. Optimizing the graft material might help in preventing subsequent keratin build‐up in the subglottis. One study has described its pioneering experience with eight cases that received a buccal graft, and none of these patients required resurfacing procedures [13]. However, buccal lining is more challenging to work with and adds morbidity to the gingival buccal sulcus. Ideally, graft material would have ciliary clearance capacity, and laboratory‐grown respiratory epithelium is a direction to explore.

We hope the results of this and previous studies [11, 12, 13] will encourage surgeons to consider the Maddern procedure in patients with idiopathic subglottic stenosis and publish on their results to substantiate the efficacy of this procedure.

## 5 Conclusion

The Maddern procedure is an effective and durable option for patients with idiopathic subglottic stenosis seeking more permanent surgical management other than repeated endoscopic dilations.

## Conflicts of Interest

The authors declare no conflicts of interest.

## Data Availability

Data available on request from the authors. The data that support the findings of this study are available from the corresponding author upon reasonable request.
